# Colour Vision Deficits in Children with Amblyopia: Impact of Angular Size of Stimuli on Detection

**DOI:** 10.3390/vision9010003

**Published:** 2025-01-10

**Authors:** Kristine Kalnica-Dorosenko, Anzelika Litavnieka, Renars Truksa, Aiga Svede, Sandra Valeina

**Affiliations:** 1 Eye Diseases Clinic, Children’s Clinical University Hospital, Vienibas Gatve 45, LV-1004 Riga, Latvia; 2 Department of Optometry and Vision Science, Faculty of Science and Technology, University of Latvia, Jelgavas Street 1, LV-1004 Riga, Latvia

**Keywords:** amblyopia, children, colour vision, visual deficits, stimulus size, computerized colour vision test

## Abstract

This study investigates colour vision deficits in children with amblyopia by employing a computerized colour vision test with varying stimulus sizes (1°, 2°, and 3°). The aim is to delineate the impact of amblyopia on colour discrimination in children and to determine the effectiveness of the computerized colour vision test in detecting these deficits. The study involved 40 participants, divided into 20 children with amblyopia and 20 without amblyopia (control group). Our findings reveal that, during binocular viewing, children with amblyopia exhibit significant impairments in colour vision both for red–green and blue–yellow axes, primarily for 1° chromatic stimuli, but not for larger stimuli (2° and 3°). These findings offer valuable insight into the functional visual limitations in pediatric amblyopia, potentially guiding more targeted clinical assessments and interventions.

## 1. Introduction

Amblyopia, recognized as the primary etiology of visual impairment among pediatric and young populations, is characterized by unilateral or bilateral diminution of visual acuity without discernible ocular pathologies [1]. Susceptibility factors for amblyopia are categorized into ocular and non-ocular domains. Ocular factors include refractive anomalies such as myopia, hyperopia, and astigmatism, as well as conditions like strabismus and anisometropia, which significantly contribute to the manifestation and development of amblyopia [2,3,4]. Non-ocular factors, including a family history of amblyopia and premature birth, also play a role [1,5,6].

The effective prevention and management of amblyopia hinge on the early identification of these predisposing factors [5]. Vision screening programmes for young children are not just crucial, but a responsibility in detecting those at risk [7]. Once diagnosed, treatment typically involves patching the stronger eye to encourage using the weaker eye, atropine eye drops, and vision therapy exercises designed to enhance visual skills and processing. The critical period for effective intervention is during early childhood, as the visual system is still developing and is more plastic [7]. However, research has shown that in adults, it is possible to improve visual acuity in the amblyopic eye, challenging the traditional notion of a rigid critical period [8,9].

Failure to detect and address amblyopia within this critical developmental window can lead to irreversible visual impairment [10]. However, with timely detection and treatment, the potential for normal visual outcomes is high. Untreated amblyopia can result in permanent vision deficits in the affected eye, preventing it from achieving normal visual acuity. This underscores the importance of routine eye examinations for children and timely treatment for those diagnosed with amblyopia to ensure optimal visual outcomes and prevent lifelong vision impairment [3]. It also highlights the crucial role of public health initiatives in prioritizing early screening and intervention to mitigate the long-term impact of amblyopia on visual health.

### 1.1. Importance of Early Detection and Intervention

Amblyopia is primarily identified by a reduction in visual acuity; however, it can also present with several other visual impairments. These impairments may include deficient accommodation [11,12], binocular dysfunction [13,14], suppression [15], instability of fixation [16,17,18], atypical contour interactions [19], positional uncertainty [20], reduced contrast sensitivity [21,22,23], spatial distortions [24], and abnormal ocular movements [25]. Amblyopia can significantly affect quality of life due to its influence on an individual’s capacity to engage in sports and physical activities, participate in social interactions, and pursue their desired career paths. Furthermore, amblyopia may lead to psychological repercussions such as depression and anxiety in affected individuals [26,27,28].

### 1.2. Importance of Colour Vision in Daily Activities

Colour vision is an integral component of visual processing, aiding in the performance of various visual tasks. It enhances informational capabilities, not only serving to perceive colours, but also contributing to the perception of form, depth, and motion [29]. Individuals with normal colour vision are called trichromats. They can accurately distinguish a wide range of hues and saturations, specifically red, blue, and green. Colour perception defects can occur due to the malfunction of one, two, or even all types of cones, and in some cases, abnormalities in post-receptoral processing may contribute to these deficits [30,31,32,33]. There are three types of colour vision deficiencies: protanomaly (reduced sensitivity to red), deuteranomaly (reduced sensitivity to green), and tritanomaly (reduced sensitivity to blue).

Colour vision deficiencies impact numerous aspects of life, from childhood to adulthood [34]. These effects permeate various domains, including recreational activities such as play and sports, for which accurate colour perception is essential [35]. In educational settings, colour vision deficiencies can hinder learning and comprehension, particularly in subjects that rely on colour-coded materials. Daily activities, such as distinguishing between different coloured objects or reading colour-coded instructions, can also be challenging. Moreover, individuals with colour vision deficiencies may face discrimination and social challenges due to their condition. Health and safety issues also arise, as the inability to perceive specific colours can affect one’s ability to interpret warning signals, traffic lights, and other critical visual cues. Additionally, individuals with colour vision deficiencies may face restrictions in pursuing certain occupations that require accurate colour discrimination, such as operating commercial vehicles, working as railroad engineers, or piloting aircraft [36]. The pervasive influence of colour vision deficiencies underscores the need for awareness and accommodation in various spheres of life.

### 1.3. Colour Vision Deficits in Children with Amblyopia

Colour vision defects are common in cases of amblyopia, especially when it is severe and monocular vision is present [37]. Amblyopia often results in spatial vision impairments, but its impact on colour discrimination remains less clearly understood, particularly in children. Previous studies on colour vision deficits in amblyopia have yielded mixed results, largely due to methodological variations in stimulus size and testing protocols. For example, in a study by Suliman and Ali, the presence of amblyopia was found to significantly impair colour vision, a finding that has important implications for our understanding of this condition. Among 64 participants with amblyopia, 39% were found to have colour vision deficiencies, a significant proportion. Specifically, nearly 28% demonstrated tritanomaly (blue colour perception defect), 6% had deuteranomaly (green colour perception defect), and 5% had protanomaly (red colour perception defect) [38]. Kocak-Altintas and colleagues demonstrated in their study that amblyopia affects colour vision, but this is not related to visual acuity or the type of amblyopia. The mistake rate in colour recognition was higher among participants with strabismic amblyopia than those with anisometropic amblyopia, though this difference was not statistically significant. However, a statistically significant difference was observed between the amblyopia and control groups [39]. In a study by Rajavi and colleagues, colour perception problems were explicitly found in children with a visual acuity of 0.5 decimal units or less. This finding has important implications for the diagnosis and management of amblyopia, as it informs us that colour vision deficiency is associated with lower visual acuity and amblyopia, but there is no correlation between colour vision deficiency and the type of amblyopia [40].

### 1.4. Colour Vision Assessment Tests

Several tests can be used to diagnose colour vision deficiencies. The most commonly used colour vision assessment tests across populations are the Farnsworth–Munsell and Ishihara tests [41]. The Farnsworth–Munsell 100 Hue test is extensively used to measure hue discrimination and identify various acquired and congenital colour vision deficiencies. It is widely recognized as one of the most sensitive and accurate tests of chromatic discrimination suitable for broad clinical application [39]. The test requires arranging colour caps in the correct order based on hue. Test results are based on the number of incorrectly placed colour caps and the degree or distance of misplacement of colour caps [42].

The Ishihara test is a colour perception test that aids in determining confusion sensitivity to red and green colours. This test belongs to the class of colour vision tests known as pseudoisochromatic plates. The examination consists of multiple Ishihara plates depicting circles with coloured dots that are presented randomly in terms of colour and size. Within the plates are dots forming a number or shape that is visible to individuals with normal colour vision but may be mis-read or impossible to discern for those with red–green colour vision deficiency [41]. Choi and Hwang in 2009 identified in their study that colour vision testing in young children poses a challenge for clinicians, as many tests require a child’s patience and attention [43]. It is important to note that children younger than five years often produce false positive results, highlighting the need for caution and thoroughness in our testing.

While numerous colour vision assessment tests are in use, the advantages of computerized versions are increasingly becoming apparent [44]. Several new versions of tests designed for colour vision assessment on computer screens have been developed and widely distributed in recent years. These tests offer a range of benefits, such as the ability to present stimuli in various sizes, colours, and saturations [45,46,47]. This flexibility is crucial for determining the form and degree of colour vision deficiency [48]. Notably, individuals with colour vision deficiency perceive more saturated colours with larger angular sizes than those with normal colour perception, a distinction that can be effectively captured by computerized tests [47]. The advent of computerized testing has revolutionized the field, offering unparalleled convenience. It can display impractical colours that are difficult to print and show on paper, facilitate easy randomization, and automatically evaluate stimuli. However, it is important to note that such assessments must explicitly specify hardware and software settings [44]. The computerized colour vision test is a robust tool for assessing colour vision because it isolates colour contrast from luminance contrast, providing precise measurements of colour discrimination thresholds. The present study uses the computerized colour vision test to explore colour vision impairments in children with amblyopia, specifically by examining the effect of varying stimulus sizes (1°, 2°, and 3°).

## 2. Materials and Methods

### 2.1. Ethical Consideration

All procedures followed the Declaration of Helsinki, and were approved by the Ethics Committee of the Experimental and Clinical Medicine Institute of the University of Latvia, Riga, Latvia. Informed consent was obtained from all the subjects involved in the study.

### 2.2. Participants

**Figure 1:**
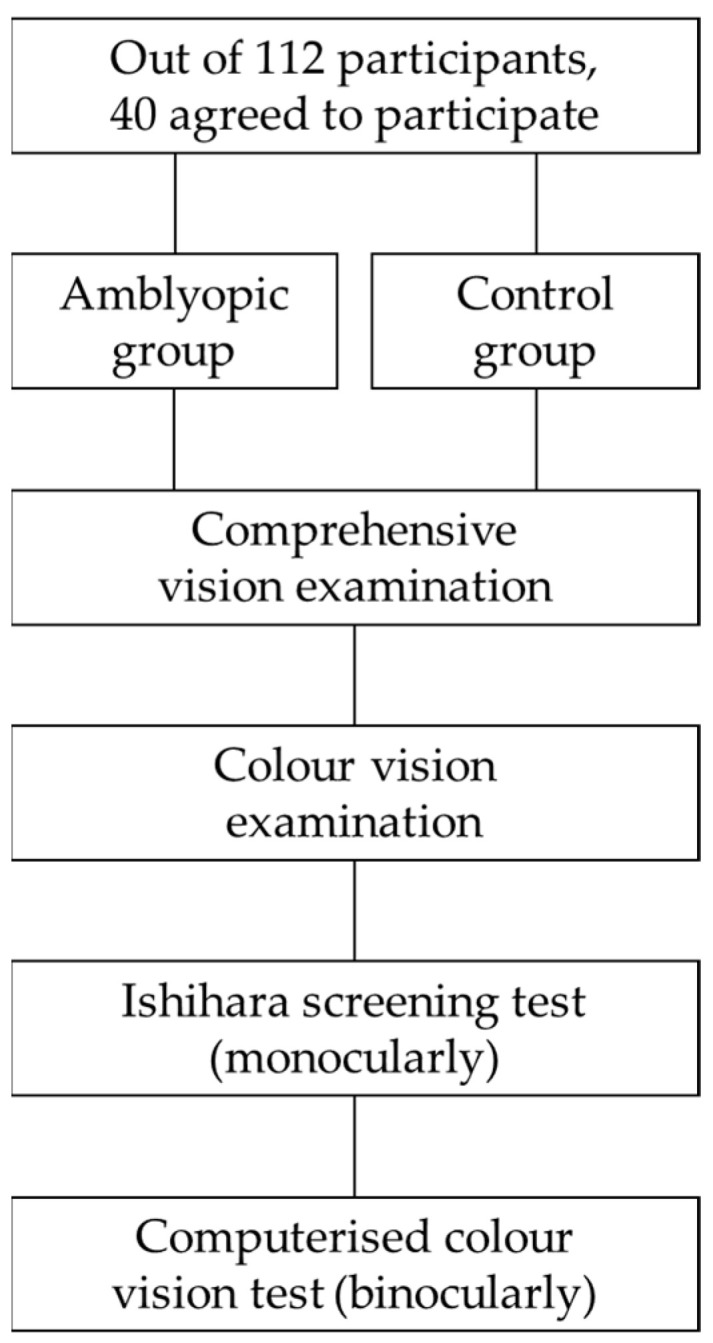
A diagram showing a brief description of the experiment.

In total, 40 children, with an average age of 8 ± 1 years (ranging from 6 to 9 years), agreed to participate in the experiment out of 112 participants from the Children’s Clinical University Hospital in Riga, Latvia. The participants were divided into two groups: individuals diagnosed with amblyopia (the difference in best-corrected visual acuity between the two eyes was ≥2 optotype lines), of whom 3 had anisometropic amblyopia (the difference in refractive error between the two eyes was ≥1 D), 7 had refractive amblyopia (an uncorrected refractive error in one eye), and 10 had strabismic amblyopia; and a control group (see Figure 1). The participants in the control group had to have a visual acuity of 1.0 or higher in decimal units. All participants were generally and neurologically healthy. Each participant underwent a comprehensive ophthalmic examination, including visual acuity testing and standard refractive assessments.

All the study participants underwent colour vision assessment using screening Ishihara and computerized colour vision tests. The computerized colour vision test was administered binocularly, while the Ishihara test was administered monocularly. The study focused on measuring colour discrimination thresholds along the red–green (RG) and blue–yellow (BY) axes using the computerized colour vision test. Stimulus sizes of 1°, 2°, and 3° were used in separate trials to observe the impact of eccentricity and spatial summation on colour discrimination in amblyopic eyes compared to non-amblyopic controls.

### 2.3. Ishihara Screening Test

The Ishihara test, a tool known for its efficiency, comprises coloured plates with various-sized spots arranged randomly to form numbers. People with normal colour vision can see these numbers, but they are hard to distinguish for those with red–green colour deficiency. The test consists of 17 plates and is administered in daylight, with each plate positioned 40 cm away from the participant. The numeral plates are divided into five design categories: 1. Demonstration (plate 1); 2. Transformation (plates 2–9); 3. Vanishing (plates 10–17) [49]. Each plate is shown once to each eye. The sequence of plates shown to each participant is randomly selected and non-repetitive. The main task for each participant is to identify the number seen on each plate. The Ishihara test is used to perform a quick screening assessment, emphasizing its role in providing rapid results. The Ishihara test was conducted monocularly, first for the amblyopic eye and then for the better-seeing eye. This order was maintained to eliminate any potential learning bias, as testing the better-seeing eye first could provide the subject with information that might influence responses during subsequent testing of the amblyopic eye. All participants in the experiment responded correctly without making a single error.

### 2.4. The Computerized Colour Vision Test

**Figure 2:**
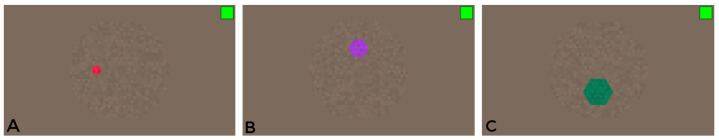
The targets for the computerized colour vision test stimuli were as follows: (**A**) a chromatic target of 1 degree along the protanopia confusion lines, (**B**) a chromatic target of 2 degrees along the tritanopia confusion lines, and (**C**) a chromatic target of 3 degrees along the deuteranopia confusion lines.

During the study, a computerized colour vision test was developed to identify colour vision deficiencies in children with and without amblyopia based on the angular size of the stimulus. The stimulus for this study was modified to adjust the target stimulus’s angular size (see Figure 2).

The visual stimuli were shown on a MacBook Pro monitor with a resolution of 2560 × 1600 pixels. To ensure an accurate display of the coloured targets, the monitor underwent calibration using a PR-655 spectroradiometer and the accompanying SpectraWin 2 software. The RGB coordinates for each stimulus were automatically determined using colour calibration data and an algorithm.

The coefficients for the linear equations were calculated based on the intersection of the confusion lines and the white point coordinates of the computer monitor (xb = 0.3063, yb = 0.3198) [48]. In order to evaluate colour vision in amblyopia patients, measurements were taken in six directions within the colour space, using three different-sized chromatic targets: 1, 2, and 3 degrees. These target sizes were chosen for their practicality, as they could be effectively displayed in the test and provided significant visual differentiation. Targets smaller than 1 degree or larger than 4 degrees could not be included in the test due to the inability to form a hexagonal shape, and the inability to determine the direction for a 4-degree target due to its excessive size.

By setting the luminance values of the test stimulus and the background elements at 40, 43, 46, 49, and 52 cd/m^2^ [50], we ensured no contrast noise. These different luminance levels play a crucial role in determining whether an individual truly perceives the colour of the target stimulus rather than its brightness or the boundaries of the target. The computerized colour vision test automatically adjusts the luminance levels, effectively masking the contrast and highlighting the importance of our methodology.

Monocular testing of colour vision remains critical in evaluating amblyopic eyes, as subtle interocular differences may not be detectable under binocular conditions. Such differences, while absent in the Ishihara test results in our study, could provide insights into visual processing alterations in amblyopia. This rationale underscores the initial utility of monocular Ishihara testing, regardless of the subsequent adaptation of the computerized test to accommodate the practical limitations of testing young children.

All measurements were taken with both eyes at a distance of 90 cm from the monitor. The angular subtense of the screen was calculated to ensure consistency in the visual presentation of stimuli. The formula used was $\alpha =2atg\left(\frac{h}{2d}\right)$, where *h* represents the screen dimension in centimetres, and *d* represents the viewing distance in centimetres. Based on this formula, the vertical angular size of the screen was approximately 13.46°, and the horizontal angular size was approximately 19.18° at the specified viewing distance. Colour stimuli were presented at an eccentricity of 2.22° from the centre of the screen. The distance was measured as the angular separation between the centre of the chromatic stimulus and the centre of the background, ensuring consistent placement across trials.

To define the confusion lines, we used linear equations characterized by their slope (*k*) and intercept (*b*) with the *y*-axis. These coefficients were calculated based on the assumption that all confusion lines pass through the monitor’s white point (*x*_w_, *y*_w_) and based on the specific confusion point characteristic of each type of colour vision deficiency: protan (*x*_p_, *y*_p_), deutan (*x*_d_, *y*_d_), and tritan (*x*_t_, *y*_t_). The formulas for the slope (*k*) and intercept (*b*) are as follows:

$$k=\frac{y_{confusion}-y_{w}}{x_{confusion}-x_{w}}\;\mathrm{and}\;b=y_{w}-{kx}_{w}$$

Substituting the coordinates of the confusion and white points, the coefficients for each confusion line were calculated as follows:

- Protan confusion line:

$$\begin{array}{c} k_{p}=\frac{y_{p}-y_{w}}{x_{p}-x_{w}}=\frac{0.25-0.3199}{0.75-0.3063}=-0.1575\\ b_{p}=y_{w}-{k_{p}x}_{w}=0.3199-(-0.1575\times 0.3063)=0.3681 \end{array}$$

- Deutan confusion line:

$$\begin{array}{c} k_{d}=\frac{y_{d}-y_{w}}{x_{d}-x_{w}}=\frac{-0.40-0.3199}{1.4-0.3063}=-0.6582\\ b_{d}=y_{w}-{k_{d}x}_{w}=0.3199-(-0.6582\times 0.3063)=0.5215 \end{array}$$

- Tritan confusion line:

$$\begin{array}{c} k_{t}=\frac{y_{t}-y_{w}}{x_{t}-x_{w}}=\frac{0-0.3199}{0.17-0.3063}=2.3467\\ b_{t}=y_{w}-{k_{t}x}_{w}=0.3199-(2.3467\times 0.3063)=-0.3989 \end{array}$$

The resulting linear equations for the protan, deutan, and tritan confusion lines are as follows:

Protan: *y* = $k_{p}x+b_{p}$

Deutan: *y* = $k_{d}x+b_{d}$

Tritan: *y* = $k_{t}x+b_{t}$

The participants in the experiment had to quickly indicate the direction of the target placement, while the chromatic hexagonal targets were displayed in four possible directions: up, down, right, or left. If no response was given during the experiment, it was counted as incorrect [51]. Approximately 20 trials were conducted for each stimulus size along each colour axis. This number varied slightly depending on the accuracy of participant responses, as determined by the adaptive procedure. Stimulus presentations were randomized using a multiple random staircase method, ensuring balanced exposure to all directions (protan, deutan, and tritan) until the required number of thresholds were measured for each. The 3 s stimulus duration and the subsequent 3 s response window were designed to balance participant accuracy and timing consistency, ensuring reliable data collection across trials. The average duration of the computerized colour vision test for each participant was 25 ± 3 min.

## 3. Results

To confirm the presence of normal colour vision, each participant underwent the Ishihara test before the computerized colour vision test. All participants provided correct responses on the Ishihara test, indicating no red–green colour vision defects.

**Figure 3:**
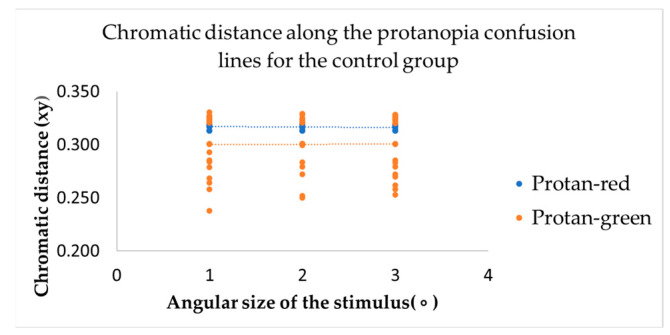
The chromatic distances along the protanopia confusion lines varied depending on the angular size of the target stimulus across the entire control group.

**Figure 4:**
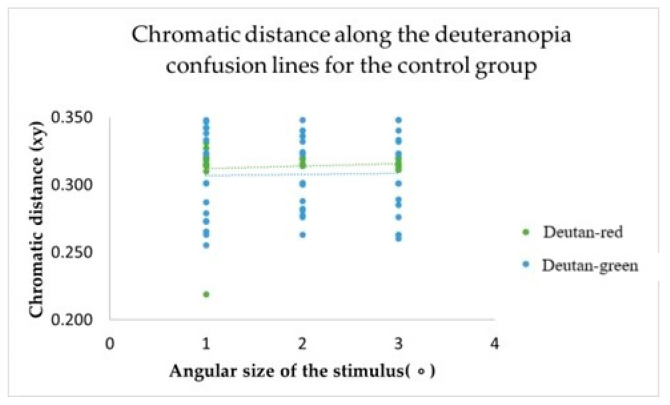
The chromatic distances along the deuteranopia confusion lines varied depending on the angular size of the target stimulus across the entire control group.

**Figure 5:**
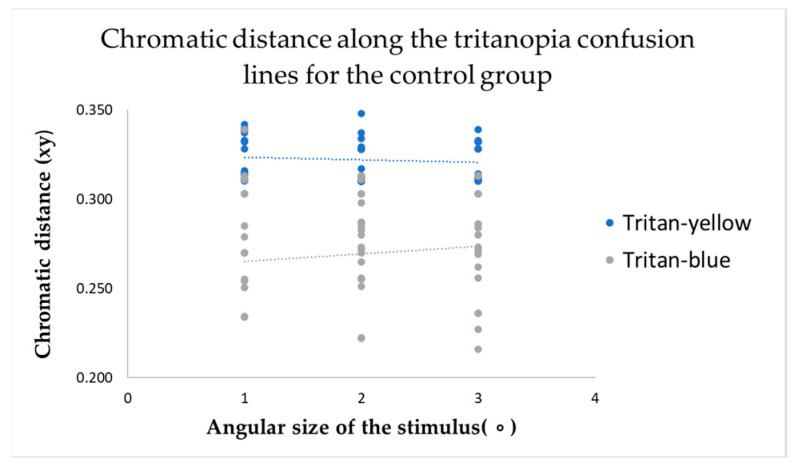
The chromatic distances along the tritanopia confusion lines varied depending on the angular size of the target stimulus across the entire control group.

The study’s primary objective was to determine whether changes in the angular size of the target stimulus have any effect on colour vision. Results from the computerized colour vision test indicated that, for the control group, variations in the angular size of the target stimulus did not alter chromatic discrimination (see Figure 3, Figure 4 and Figure 5).

**Figure 6:**
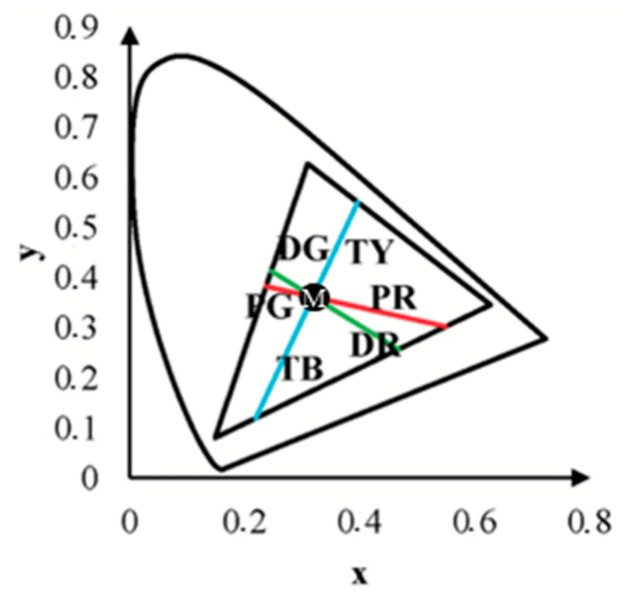
The CIExy diagram illustrates the colour direction codes along the protan, deutan, and tritan confusion lines, specifically PR-protan-red, PG-protan-green, DG-deutan-green, DR-deutan-red, TB-tritan-blue, and TY-tritan-yellow. The monitor’s white point, represented as a black point in the diagram, is chosen at coordinates (0.3188, 0.36). The confusion lines were selected to include the corresponding confusion points: protan (0.75, 0.25), deutan (1.4, −0.4), tritan (0, 0.17), and the monitor’s white point [45].

The chromatic distance, a crucial parameter in our study, was obtained from the test results for the confusion line directions’ *x* and *y* coordinates, at *xy*-distance from the white point (See Figure 6).

This metric determines the distance of confusion lines from the white point—more considerable chromatic distances correspond to a more significant number of incorrect responses in determining the orientation of the chromatic stimulus, indicating the presence of colour vision deficiency. The mean value of chromatic distance for the control group, regardless of the angular size of the target stimulus, for the protan-red confusion line was 0.32 xy, for protan-green was 0.30 xy, for deutan-red and deutan-green was 0.31 xy, for tritan-yellow was 0.32 xy, and for tritan-blue was 0.28 xy, indicating that for each confusion line, chromatic distance remains unchanged, irrespective of the angular size of the target stimulus. These findings significantly impact our understanding of colour vision deficiency, potentially leading to new diagnostic and treatment methods. The linear trend curve supports this.

Our research involved a thorough statistical analysis, comparing the chromatic distances for each confusion line in the control and amblyopia groups across three angular sizes of the target stimulus. The results for the control group were consistent, with all targets, regardless of angular size, being consistently identified along all confusion lines. Multivariate Analysis of Variance (MANOVA) was performed to examine chromatic difference thresholds along the six colour-confusion axes (protan-red, protan-green, deutan-red, deutan-green, tritan-yellow, and tritan-blue) as a function of angular stimulus size. This statistical method accounted for potential interactions between stimulus size and chromatic thresholds across multiple axes. A Multivariate Analysis of Variance (MANOVA, one-way) test further demonstrated that for the protan-red (F(2,117) = 0.59; *p* = 0.56), protan-green (F(2,117) = 0.16; *p* = 0.85), deutan-red (F(2,117) = 1.57; *p* = 0.21), deutan-green (F(2,117) = 0.36; *p* = 0.82), tritan-yellow (F(2,117) = 0.50; *p* = 0.47), and tritan-blue (F(2,117) = 0.36; *p* = 0.36) confusion lines, there were no statistically significant differences (*p*-value: *p* > 0.05) when varying the angular sizes of the target stimulus in the computerized colour assessment test. Multivariate Analysis of Variance (MANOVA) is a statistical technique that extends the functionality of Analysis of Variance (ANOVA) by enabling the simultaneous analysis of multiple continuous dependent variables. Most responses correctly identified the direction of the chromatic target despite the reduction in the target stimulus’s angular size. The test results did not indicate any colour vision deficiencies in the control group.

Post hoc analysis tests indicated no statistically significant differences in chromatic distances along the confusion lines with variation in the angular sizes of the target stimulus, as the *p*-value for each comparison was greater than 0.05 (*p*-values for pairwise comparisons between target sizes for the control participants ranged from 0.209 to 0.999).

**Figure 7:**
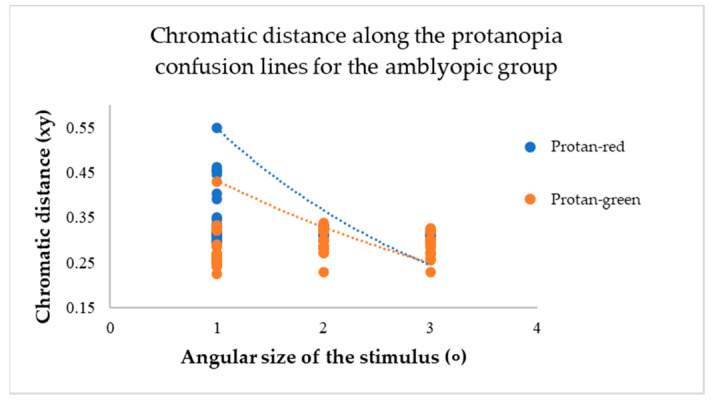
The chromatic distances along the protanopia confusion lines varied depending on the angular size of the target stimulus across the entire amblyopic group.

**Figure 8:**
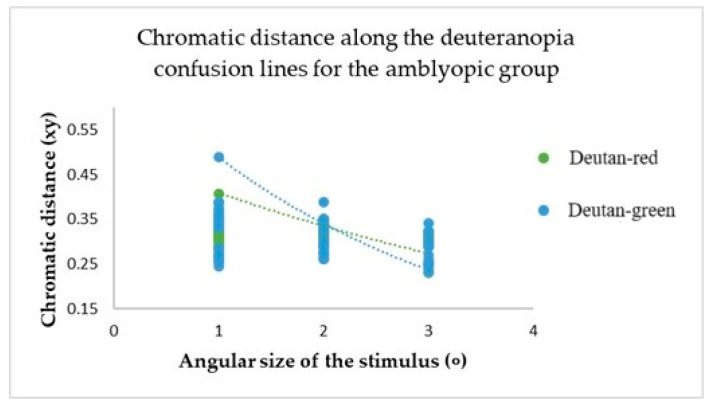
The chromatic distances along the deuteranopia confusion lines varied depending on the angular size of the target stimulus across the entire amblyopic group.

**Figure 9:**
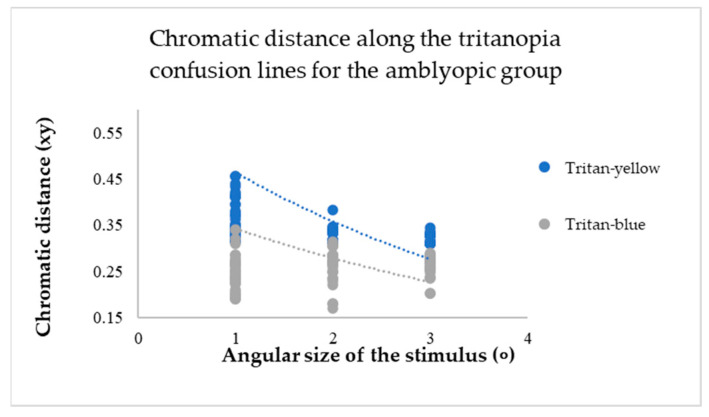
The chromatic distances along the tritanopia confusion lines varied depending on the angular size of the target stimulus across the entire amblyopic group.

For participants in the amblyopic group, a decrease in the angular size of the target resulted in an increase in chromatic distance for each confusion line (see Figure 7, Figure 8 and Figure 9).

The mean values of chromatic distance indicate this. For instance, for the protan-red confusion line (see Figure 7), the mean chromatic distance value at larger target angular sizes 2 and 3 degrees is 0.32 xy. However, the mean chromatic distance value increases to 0.47 xy at the smallest angular size. This indicates that as the target angular size decreases, it becomes more challenging for the amblyopia group to determine the target’s direction. Thus, the computerized test results reveal a colour vision deficiency. The same trend is observed for the deuteranopia and tritanopia confusion lines (see Figure 8 and Figure 9).

A Multivariate Analysis of Variance (MANOVA, one-way) test showed that as the target angular size changed, the chromatic distance for each confusion line also changed, and these differences were statistically significant (*p*-value: *p* < 0.05). Specifically, the results were significant for the protan-red (F(2,117) = 7.50; *p* < 0.001), deutan-red (F(2,117) = 3.29; *p* < 0.001), deutan-green (F(2,117) = 9.88; *p* < 0.001), tritan-yellow (F(2,117) = 32.31; *p* < 0.001), and tritan-blue (F(2,117) = 5.06; *p* < 0.001) confusion lines. The protan-green confusion line (F(2,117) = 1.25; *p* = 0.19) did not show a statistically significant difference. For participants with amblyopia, a decrease in the target angular size affects colour vision (*p* < 0.05) as the chromatic distance increases, indicating a colour vision deficiency.

However, post hoc analysis tests indicated that for each confusion line, chromatic distances statistically differed when comparing specific target angular sizes (see Table 1).

When the target angular size decreased to 1 degree, the average values of chromatic distances in the test results increased, indicating a colour vision deficiency. However, for the protan-green confusion line alone, there was no statistically significant difference compared to all the angular sizes of the target (*p* > 0.05).

**Figure 10:**
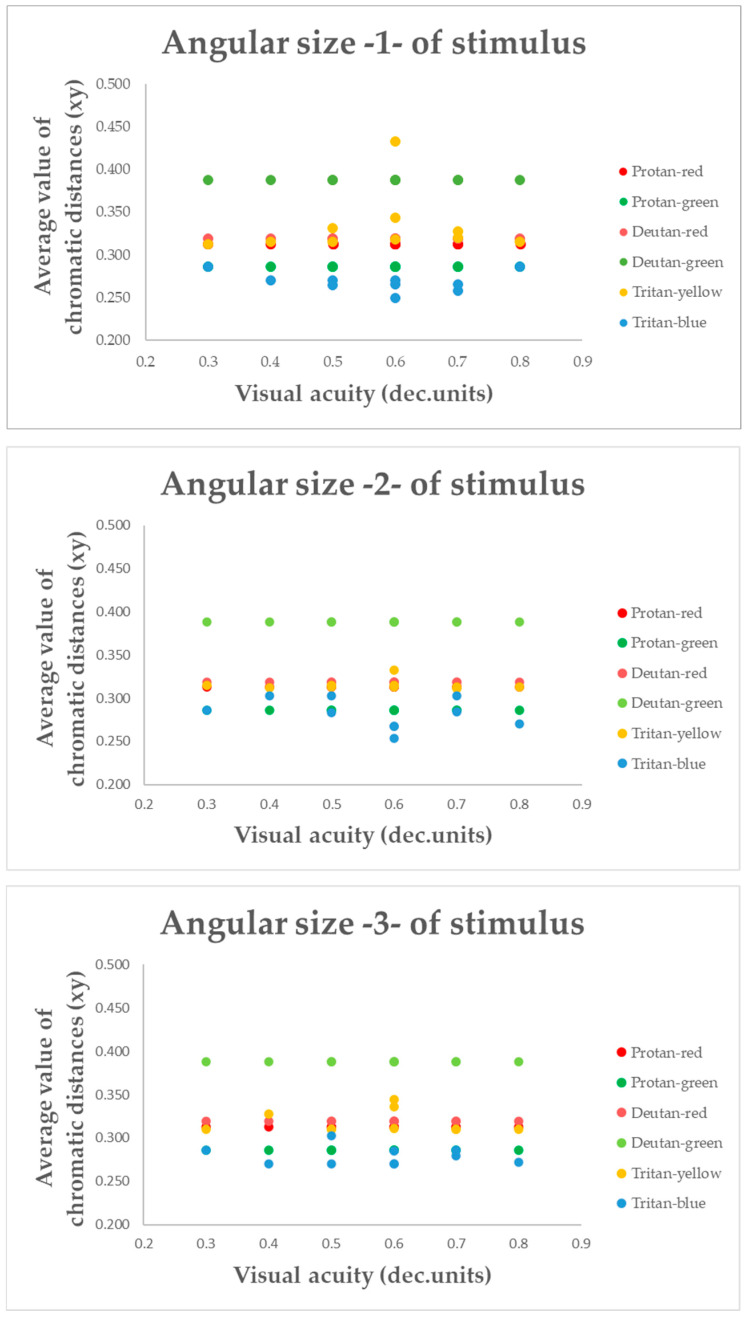
The mean chromatic distance, depending on target angular size and visual acuity (in decimal units).

To better understand whether the colour vision deficit is indeed influenced by changes in the target’s angular size, the mean chromatic distance for each confusion line in participants with amblyopia was compared not only with the target’s angular size, but also with visual acuity (see Figure 10).

Figure 10 shows that the average chromatic distance value changes with each level of visual acuity, but no significant increase or decrease in the average value is observed at any specific level of visual acuity. The chromatic distance remains constant and linear for each target angular size, regardless of visual acuity. The results of a two-way ANOVA indicated that visual acuity had no statistically significant effect on the chromatic distance along the confusion lines (*p* > 0.05). Therefore, changes in visual acuity did not affect the recognition of confusion lines in the amblyopia group.

## 4. Discussion

All the studies reviewed in this work related to amblyopia and colour vision presented varied conclusions and results. Consequently, it remains unclear precisely what influences colour vision in children with amblyopia and what factors might be associated with this condition. For example, Rajavi et al. [40] determine that colour vision deficiency arises specifically due to reduced visual acuity, whereas Suliman and Ali [38] suggest that it depends on the degree of amblyopia. Our initial hypothesis was confirmed, aligning with the conclusions of Kocak-Altintas et al. [39], which indicate that colour vision deficiency is not associated with visual acuity or the type of amblyopia.

Our findings suggest that children with amblyopia experience significant colour vision deficits, which are influenced by the spatial properties of the stimulus. Smaller stimulus sizes reveal more pronounced deficits, while larger stimuli mitigate these effects to a degree. The dependency on stimulus size highlights the importance of spatial summation and retinal coverage in chromatic discrimination. Given that amblyopes are characterized by abnormal binocularity, suppression, and non-foveal viewing [13,14,15,16,17,26] in the amblyopic eye, these findings likely reflect processing in the non-amblyopic eye during binocular viewing. It is possible that more profound colour vision deficits would be observed if testing were conducted monocularly using the amblyopic eye. Additionally, the computerized colour vision test’s ability to isolate colour contrast from luminance allows for a more accurate characterization of these deficits. It provides a robust tool for future investigations of monocular colour processing in amblyopia.

In future studies, it is recommended to reduce the testing time of the computerized colour vision test, as the colour vision assessment currently requires an average of 25 min per child, with each target angular size taking approximately 8 min. This duration strains the child’s ability to maintain focus, increasing the likelihood of inaccurate results.

## 5. Conclusions

This study provides novel insights into the colour vision deficits associated with amblyopia in children, illustrating that stimulus size impacts colour discrimination ability. These results underscore the value of using variable stimulus sizes in clinical colour vision assessments, and suggest that the computerized colour vision test is a sensitive tool for detecting colour deficits in pediatric amblyopia. Future research should explore longitudinal changes in colour vision with amblyopia treatment to assess the potential reversibility of these deficits.

## Figures and Tables

**Table 1:** The *p*-values comparing chromatic distances along the confusion lines for each target angular size were calculated for the amblyopic group.

	Angular Size of the Stimulus	Comparison of Angular Stimulus Size	*p*-Value
Protan-red	1	2	0.004
		3	0.002
	2	1	0.004
		3	0.983
	3	1	0.002
		2	0.983
Protan-green	1	2	0.285
		3	0.501
	2	1	0.285
		3	0.917
	3	1	0.501
		2	0.917
Deutan-red	1	2	0.260
		3	0.033
	2	1	0.260
		3	0.601
	3	1	0.033
		2	0.601
Deutan-green	1	2	0.206
		3	<0.001
	2	1	0.206
		3	0.022
	3	1	<0.001
		2	0.022
Tritan-yellow	1	2	<0.001
		3	<0.001
	2	1	<0.001
		3	0.341
	3	1	<0.001
		2	0.341
Tritan-blue	1	2	0.017
		3	0.020
	2	1	0.017
		3	0.998
	3	1	0.020
		2	0.998

## Data Availability

The original contributions presented in this study are included in the article. Further inquiries can be directed to the corresponding author.

## References

[1] Pai A.S.I., Rose K.A., Leone J.F., Sharbini S., Burlutsky G., Varma R., Wong T.Y., Mitchell P. (2012). Amblyopia prevalence and risk factors in Australia preschool children. Ophthalmology.

[2] Robaei D., Rose K.A., Ojaimi E., Kifley A., Martin F.J., Mitchell P. (2006). Causes and associations of amblyopian a population-based sample of 6-years-old Australian children. Arch. Ophthalmol..

[3] Williams C., Northstone K., Howard M., Harvey I., Harrad R.A., Sparrow J.M. (2008). Prevalence and risk factors for common vision problems in children: Data from ALSPAC study. Br. J. Ophthalmol..

[4] Cumberland P.M., Pathai S., Rahi J.S., Millennium Cohort Study Child Health Group (2010). Prevalence of eyedisease in early childhood and associated factors: Findings from the Millennium Cohort Study. Ophthalmology.

[5] Mocanu V., Horhat R. (2018). Prevalence and risk factors of amblyopia among refractive errors in an Eastern European population. Medicina.

[6] Hakima R.B., Tielsch K.M. (1992). Maternal cigarette smoking during pregnancy: A risk factor for childhood strabismus. Arch. Ophthalmol..

[7] Birch E.E., Kelly K.R., Wang J. (2021). Recent advances in screening and treatment for amblyopia. Ophthalmol. Ther..

[8] Jeon S.T., Maurer D., Lewis T.L. (2012). The effect of video game training on the vision of adults with bilateral deprivation amblyopia. Seeing Perceiving.

[9] Žiak P., Holm A., Halička J., Mojžiš P., Pińero D.P. (2017). Amblyopia treatment of adults with dichoptic training using the virtual reality oculus rift head mounted display: Preliminary results. BMC Ophthalmol..

[10] Parks M.M., Duane T.D. (1989). Treatment of the sensorial adaptations and amblyopia. Clinical Ophthalmology.

[11] Ale Magar J.B., Shah S.P. (2022). Accommodative lag persistence in treated anisometropic, strabismic, and mixed amblyopia. J. Ophthalmol..

[12] Chen A.M., Manh V., Candy T. (2018). Longitudinal evaluation of accommodation during treatment for unilateral amblyopia. Ophthalmol. Vis. Sci..

[13] Birch E.E. (2013). Amblyopia and binocular vision. Prog. Retin. Eye Res..

[14] McKee S.P., Levi D.M., Movshon J.A. (2003). The pattern of visual deficits in amblyopia. J. Vis..

[15] Li J., Thompson B., Lam C.S.Y., Deng D., Chan L.Y.L., Maehara G., Woo G.C., Yu M., Hess R.F. (2011). The role of suppression in amblyopia. Investig. Ophthalmol. Vis. Sci..

[16] Scaramuzzi M., Murray J., Otero-Millan J., Nucci P., Shaikh A.G., Ghasia F.F. (2019). Fixation instability in amblyopia: Oculomotor disease biomarkers predictive of treatment effectiveness. Prog. Brain Res..

[17] Shaikh A.G., Otero-Millan J., Kumar P., Ghasia F.F. (2016). Abnormal fixational eye movements in amblyopia. PLoS ONE.

[18] Subramanian V., Jost R.M., Birch E.E. (2013). A quantitative study of fixation stability in amblyopia. Investig. Ophthalmol. Vis. Sci..

[19] Simmers A.J., Gray L.S., McGraw P.V., Winn B. (1999). Contour interaction for high and low contrast optotypes in normal and amblyopic observers. Ophthalmic Physiol. Opt..

[20] Fronius M., Sireteanu R., Zubcov A., Buttner A. (2000). Preliminary report: Monocular spatial localization in children with strabismic amblyopia. Strabismus.

[21] Wang G., Zhao C., Ding Q., Wang P. (2017). An assessment of the contrast sensitivity in patients with ametropic and anisometropic amblyopia in achieving the corrected visual acuity of 1.0. Sci. Rep..

[22] Liao M., Zhao H., Liu L., Li Q., Dai Y., Zhang Y., Zhou Y. (2016). Training to improve contrast sensitivity in amblyopia: Correction of high-order aberrations. Sci. Rep..

[23] Zele A.J., Pokorny J., Lee D.Y., Ireland D. (2007). Anisometropic amblyopia: Spatial contrast sensitivity deficits in inferred magnocellular and parvocellular vision. Investig. Ophthalmol. Vis. Sci..

[24] Li J., Spiegel D.P., Hess R.F., Chen Z., Chan L.Y., Deng D., Yu M., Thompson B. (2015). Dichoptic training improves contrast sensitivity in adults with amblyopia. Vis. Res..

[25] Sireteanu R., Thiel A., Fikus S., Iftime A. (2008). Patterns of spatial distortions in human amblyopia are invariant to stimulus duration and instruction modality. Vis. Res..

[26] Kang S.L., Beylergil S.B., Otero-Millan J., Shaikh A., Ghasia F. (2019). Fixational eye movement waveforms in amblyopia: Characteristics of fast and slow eye movements. J. Eye Mov. Res..

[27] Aljohani M.M., Alorabi S.O., Alrajhi Z.M., Jamjoom L.H. (2018). Awareness, attitudes and practices regarding common eye diseases among general population in Saudi Arabia. Ann. Int. Med. Dent. Res..

[28] Webber A.L. (2018). The functional impact of amblyopia. Clin. Exp. Optom..

[29] Maconachie G.D.E., Gottlob I. (2015). The challenges of amblyopia treatment. Biomed. J..

[30] Simunovic M.P. (2010). Colour vision deficiency. Eye.

[31] Sharpe L.T., Stockman A., Jagle H., Nathans J., Gegenfurther K.R., Sharpe L.T. (1999). Opsin genes, cone photopigments, colour vision and colour blindness. Color Vision.

[32] Chirimuuta M., Kingdom F.A.A. (2015). The uses of colour vision: Ornamental, practical, and theoretical. Minds Mach..

[33] Bartolomeo P. (2021). Color vision deficits. Curr. Neurol. Neurosci. Rep..

[34] Wissinger B., Sharpe L.T. (1998). New aspects of an old theme: The genetic basis of human color vision. Am. J. Hum. Genet..

[35] Taore A., Lobo G., Turnbull P.R., Dakin S.C. (2022). Diagnosis of colour vision deficits using eye movements. Sci. Rep..

[36] Steward J.M., Cole B.L. (1989). What do color vision defectives say about everyday tasks?. Optom. Vis. Sci..

[37] Singh A.K., Khan M.A., Singh A., Maheshwari A. (2021). Color vision in civil aviation. Indian J. Ophthalmol..

[38] Von Norden G.K., Campos E.C. (2002). Binocular Vision and Ocular Motility.

[39] Suliman I.A.M., Ali M.S.A. (2017). The effect of amblyopia on contrast sensitivity, color vision, and stereoacuity. Al-Basar Int. J. Ophthalmol..

[40] Koçak–Altintas A.G., Satana B., Koçak I., Duman S. (2000). Visual acuity and color vision deficiency in amblyopia. Eur. J. Ophthalmol..

[41] Rajavi Z., Sabbaghi H., Baghini A.S., Yaseri M., Sheibani K., Norouzi G. (2015). Prevalence of color vision deficiency and its correlation with amblyopia and refractive errors among primary school children. J. Ophthalmic Vis. Res..

[42] Murphy R.A. (2015). Comparing Color Vision Testing Using the Farnsworth-Munsell 100-Hue, Ishihara Compatible, and Digital TCV Software. College of Optometry. http://commons.pacificu.edu/opt/9.

[43] Kinnear P.R., Sahraie A. (2002). New Farnsworth–Munsell 100 hue test norms of normal observers for each year of age 5–22 and for age decades 30–70. Br. J. Ophthalmol..

[44] Choi S.Y., Hwang J.M. (2009). Ishihara test in 3- to 6-year-old children. Jpn. J. Ophthalmol..

[45] Ng J.S., Self E., Vanston J.E., Nguyen A.L., Crognale M.A. (2015). Evaluation of the waggoner computerized color vision test. Optom. Vis. Sci..

[46] Kutas G., Gócza K., Bodrogi P., Schand J. Color size effect. Proceedings of the Second European Conference on Colour Graphics, Imaging and Vision (CGIV 2004).

[47] Xiao K.D., Luo M.R., Li C.J., Cui G.H., Park D. (2011). Investigation of colour size effect for colour appearance assessment. Color Res. Appl..

[48] Barbur J.L., Rodriguez-Carmona M., Harlow A. (2006). Establishing the statistical limits of “normal” chromatic sensitivity. CIE Proc, Expert Symposium, CIE x 030.

[49] Sosnova T.L. (2004). Quantitative criteria for the evaluation of congenital chromatic vision disorders. Gig. I Sanit..

[50] Birch J. (1997). Efficiency of the Ishihara test for identifying red-green colour deficiency. Ophthalmic Physiol. Opt..

[51] Truksa R., Jurasevska K., Livzane A., Dzenis J. (2017). Differences in chromatic sensitivity estimated using static and dynamic colour stimuli. Proc. Latv. Acad. Sci..

